# Erratum to “Evaluation of updated Feed Saved breeding values developed in Australian Holstein dairy cattle” (JDS Commun. 3:114–119)

**DOI:** 10.3168/jdsc.2022-3-5-379

**Published:** 2022-09-12

**Authors:** S. Bolormaa, I.M. MacLeod, M. Khansefid, L.C. Marett, W.J. Wales, G.J. Nieuwhof, C.F. Baes, F.S. Schenkel, M.E. Goddard, J.E. Pryce

The animal ethics statement was missing from this paper. The following statement should have been included: **Ethics approval was required and approved for Australian study and each contributing country was responsible for ethics to collect feed intake data**.
